# 

**Published:** 1896-01

**Authors:** 


### CONTENTS FOR JANUARY, 1896.

ORIGINAL COMMUNICATIONS. PAGE.

Concerning the Symptomatology and Treatment of Infantile Diarrhea at the Buffalo Fresh Air Mission Hospital. By IRVING M. SNOW, M. D. .... 449
Observations on General Paresis. By F. H. STEPHENSON, M. D. .... 457
A Broken Neck—Recovery. By GREGORY DOYLE, M. D. .... 464
Report of a Case of Appendicitis Without Preceding Pain or Fever. By S. L. ELSNER, M. D. .... 469
Recollections and Observations of a Country Practice, With Cases. By GEORGE M. PALMER, M. D. .... 479
On the Relation of Peliosis to the Other Forms of Idiopathic Purpura. By ALFRED E. DIEHL, A. B., M. D. .... 483

CLINICAL REPORT.

A Peculiar Case of Syphilitic Infection .... 484

SOCIETY PROCEEDINGS.

Medical Society of the County of Erie .... 485
Medical Department University of Buffalo .... 487

PROGRESS IN MEDICAL SCIENCE.

Genito-Urinary and Syphilitic Diseases .... 488
Pediatrics .... 492
Public Health, Hygiene and Bacteriology .... 496

SELECTION.

The Gold Combinations as Alteratives .... 497

CORRESPONDENCE.

Mydriatics in Refraction .... 501
Refraction Without Mydriatics .... 505

EDITORIAL.

The Erie County Hospital Again .... 506
Topics of the Month .... 507

Personal .... 511
Obituary .... 512
Society Meetings .... 515
Reviews .... 518
Literary Notes .... 524
Miscellany .... 526

